# Thrombocytosis after hip and knee surgery in the rehabilitation setting: is it an occasional phenomenon? Relationship with deep venous thrombosis and functional outcome

**DOI:** 10.1186/s12891-015-0550-1

**Published:** 2015-04-15

**Authors:** Domenico Intiso, Filomena Di Rienzo, Andrea Iarossi, Massimiliano Copetti, Luigi Pazienza, Mario Russo, Maurizio Tolfa, Giuseppe Maruzzi

**Affiliations:** Rehabilitation Medicine and Neuro-rehabilitation Unit, IRCCS “Casa Sollievo della Sofferenza”, Viale dei Cappuccini, 71013 San Giovanni Rotondo, FG Italy; Biostatistics Unit, IRCCS “Casa Sollievo della Sofferenza”, San Giovanni Rotondo, FG Italy

**Keywords:** Platelets, Thrombocytosis, Orthopaedic surgery, Functional outcome, Deep venous thrombosis

## Abstract

**Background:**

Thrombocytosis can follow surgery and has occasionally been observed after major orthopaedic surgery. The aim of the present study was to ascertain the platelet count (PLTC) change in patients admitted to a rehabilitation unit after major joint surgery and whether deep venous thrombosis (DVT) and poor outcomes occurred in those who had thrombocytosis.

**Method:**

PLTC, red blood cells (RBC), haemoglobin (Hb), fibrinogen, and D-dimers were assessed in patients on admission and at discharge after major joint surgery. Functional outcomes were ascertained using the Barthel Scale (BS), the Functional Independence Measure (FIM) and gait evaluation. Thrombocytosis was considered to have occurred when PLTC was greater than or equal to 500 × 100^9^/L. All subjects with thrombocytosis had ultrasonography to assess DVT occurrence. The patients were divided into “young” and “old” groups according to an age cut-off of 75 years to investigate potential age-related differences.

**Results:**

Two hundred and seventy-five patients were identified and 142 (36 M and 106 F, mean age 77.2 ± 10.7) were enrolled. Seventy-six (53.5%) underwent total hip arthroplasty (THA), 40 (51.1%) underwent hip internal fixation and 26 (18.3%) subjects underwent total knee arthroplasty (TKA). The young and old groups included 60 and 82 patients, respectively. Fifty-nine (42.4%) patients had PLTC above 400 × 100^9^/L. Of these, 28 (20.1%) had thrombocytosis with PLTC above 500 × 100^9^/L, and 15 of them (10.7%) had very high values above 600 × 100^9^/L. Increased levels of fibrinogen and D-dimers were also detected. No subject with thrombocytosis had DVT. Outcome was not affected by PLTC. At discharge, significant improvement in all functional assessments was observed in young compared to old people; gait: 2.9 ± 0.2 vs. 2.2 ± 0.8; BS: 97 ± 6.9 vs. 70.5 ± 25.6; and FIM: 116.4 ± 10.9 vs. 83.6 ± 31.2 (p < 0.004), respectively. BS and FIM mean scores were positively correlated with Hb level.

**Conclusion:**

Elevated PLTC and thrombocytosis were not uncommon in patients after major joint surgery, but no subject developed DVT. Platelet count change did not affect the outcome. Higher age and lower haemoglobin level correlated with poorer functional recovery.

## Background

A platelet count (PLTC) that is elevated above a normal value is defined as thrombocytosis. Thrombocytosis is classified according to its origin: primary thrombocytosis refers to haematological disorders; and secondary thrombocytosis refers to several pathological clinical conditions including infections [1], malignancy, chronic inflammatory diseases [2-4] and critical illnesses [5,6]. Thrombocytosis is common among patients with severe injuries admitted to intensive care units (ICU) [6] and can be associated with significantly higher rates of venous thromboembolism [7]. The aetiology and clinical significance of such elevated PLTC in patients with injuries remains unclear, even if it has been generally recognised as a reactive phenomenon. Furthermore, although severe anaemia requiring blood transfusion can occur after major orthopaedic surgery, little is known about the changes in the PLTC after these surgical interventions. Due to the risk of venous thromboembolism, prophylactic treatment with subcutaneous low-weight heparin is normally administered. However, physicians worry about the heparin-induced thrombocytopoenia that may occur during this treatment, since the condition may be life-threatening and require intervention [8]. Thrombocytosis is observed after surgery, particularly after coronary artery by-pass [9] and major abdominal operations. Abnormally elevated platelet level has also been described after orthopaedic surgery [3,10,11] either as one of several conditions involved in secondary thrombocytosis [3] or as an unexpected finding after prophylactic administration of enoxaparin [11]. Only one study reported elevated PLTC after orthopaedic surgery, but it focussed on extreme thrombocytosis [10] and the authors were doubtful that the finding was unique to that surgery. It is well known that blood parameters such as haemoglobin and albumin level can hamper the functional outcome in subjects after major joint surgery, particularly in older people [12,13]. On the other hand, no investigation has been reported addressing PLTC changes after major joint surgery. Subjects with elevated PLTC and thrombocytosis could be more likely to develop venous thromboembolism after major joint surgery and could have different outcomes. The aim of the present study was to ascertain PLTC change in patients who underwent major joint surgery and whether those with elevated platelets were prone to thrombotic events and to poor outcomes.

## Methods

Consecutive patients discharged from the orthopaedic unit and admitted to the intensive rehabilitation unit of our hospital were identified. The ward uses multidisciplinary staff and a multidisciplinary approach to treating patients. Our staff work in teams to apply management and rehabilitation strategies coupled with a comprehensive geriatric assessment aimed at preventing complications and caring for concomitant diseases. With approval from the ethics committee of our Hospital IRCCS “Casa Sollievo della Sofferenza” and after obtaining written informed consent, patients who had undergone hip and knee surgery due to severe arthritis or fracture who required surgical intervention or arthroplasty were enrolled. Those with infections, malignancy, neurological diseases, renal and liver failure, osteomyelitis, multiple trauma, rheumathoid arthritis, primary thrombocytosis or previously ascertained blood disorders were excluded because of potential confounding effects on the results.

### Clinical and functional assessment

Comorbidities and data regarding the causes of major joint surgery, including the type of orthopaedic surgery, the time elapsed after surgery before rehabilitation admission, and the length of stay were collected. Comorbidities were evaluated using the Cumulative Illness Rating Scale (CIRS) on admission [14]. A global functional evaluation was undertaken using the Barthel Scale (BS) [15] and the Functional Independence Measure (FIM) [16] on admission and at discharge. A limitation of these scales lies in the definition of functional modifications in ambulation. To offset this inadequacy, gait was independently classified into four functional levels graded from zero to three: grade zero, no gait or bedridden; grade one, uses a wheelchair; grade two, uses a double support or walker; and grade three, walks unaided or uses a cane [17]. The BS provides a quantification of the global functional recovery and dependence in some of the basic activities of self-care. It ranges from zero to 100, with zero indicating a totally dependent, bedridden state and 100 indicating that the patient is fully independent. The activities can be divided into behaviour that relates to self-care (feeding, grooming, bathing, dressing, bowel and bladder care and toilet use) and behaviour related to mobility (ambulation, transfers and stairs climbing).

Motor and cognitive FIM subscales include 18 items. Each item uses a seven-point ordinal scale from total assistance (a score of one) to complete independence (a score of seven). The motor FIM scores are divided into four subscales that include 13 items as follows: self-care (eating, grooming, bathing, upper body dressing, lower body dressing, and toileting), sphincter control (bladder management and bowel management), mobility (bed, chair and wheelchair transfer, toilet transfer, and bathtub or shower transfer), and locomotion (walking or wheelchair and ability to climb stairs).

### Assessment of platelet and blood parameters

Blood parameters including PLTC, red blood cells (RBC), haemoglobin (Hb) and albumin level were assessed on admission and at discharge. Furthermore, fibrinogen, erythrocyte sedimentation rate (ESR), and D-dimers were assessed at the same time. PLTC was evaluated using an automatic counter (Coulter LH780, Haematology Analyser, Beckman, Miami, USA). Blood samples were collected in tubes with potassium EDTA and were analysed 1 h after venipuncture. D-dimers were measured using an Elisa technique (Vidasd-dimer, Biomerieux, France).

### Degree of thrombocytosis

Thrombocytosis refers to a PLTC above the normal value. For our laboratory, the normal PLTC range was 150–380 × 100^9^/L. A PLTC was considered elevated or abnormal when the value was higher than the established normal laboratory range. The PLTC for each subject was recorded as either mildly high (381–500 × 100^9^/L), high (501–600 × 100^9^/L), or very high (>600 × 100^9^/L). Thrombocytosis was considered to be present when a PLTC was greater than or equal to 500 × 100^9^/L. All subjects with thrombocytosis underwent lower limb duplex scan ultrasonography to assess deep venous thrombosis (DVT) occurrence. The examination was repeated in those who had persistent thrombocytosis at discharge. Furthermore, in order to ascertain the difference in platelet change between old and young people, the enrolled sample was divided into young and old groups where the subjects ages were equal to or less than 75 years or more than 75 years, respectively.

### Rehabilitative interventions

All subjects received rehabilitation treatment for approximately two hours a day, six days per week in accordance with their clinical condition. Upon admission, the subjects were moved from beds to chairs. The rehabilitation program consisted of joint mobilisation, proprioceptive neuromuscular facilitation, flexibility exercises, strength exercises and gait training. An unrestricted weight-bearing regimen was initiated in those who had TKA and THA. Subjects with dynamic compression hip screws with a plate received a gradually increasing load on the affected limb.

### Statistical analysis

Longitudinal measures were analysed using repeated measurements ANOVA. Between groups comparisons were performed using the Mann–Whitney *U* test or unpaired two-tailed Student’s *t*-test at each time point. The Pearson chi-square test was used to compare categorical variables. For the ANOVA analyses, values missing for a patient were linearly interpolated. Correlations between continuous variables were assessed using the Spearman coefficient. A p-value < 0.05 was considered as statistically significant. All analyses were performed using SAS Release 9.3 statistical software.

## Results

**Figure 1:**
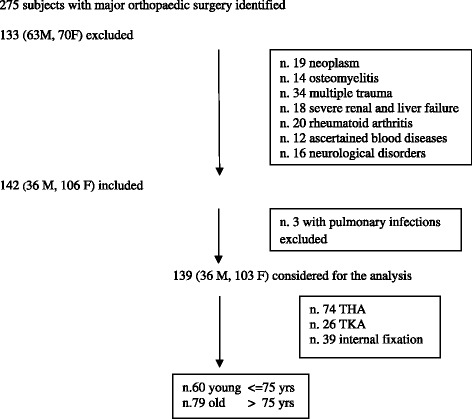
Flow chart. Legend: THA = total hip arthroplasty; TKA = total knee arthroplasty.

Two hundred and seventy-five subjects were identified. Among these, 133 (63 male and 70 female, mean age 68.4 ± 9.3) subjects were excluded (19 due to neoplasm, 34 due to multiple trauma, 14 due to osteomyelitis, 18 due to severe renal and liver failure, 20 due to rheumatoid arthritis, 12 due to ascertained blood diseases and 16 subjects due to neurological disorders), and 142 subjects were enrolled: 36 males and 106 females, mean age 77.2 ± 10.7 (range: 52–99) (Figure 1). Of the enrolled sample, 60 (15 male and 45 female; mean age 66.9 ± 6.2, range 52–75) and 82 (21 male and 61 female; mean age 84.7 ± 6.0, range 76–99) subjects were entered in the young and old groups, respectively. Seventy-six (53.5%) underwent total hip arthroplasty (THA), 40 (51.1%) underwent hip internal fixation and 26 (18.3%) subjects underwent total knee arthroplasty (TKA). Hip fracture was prevalent in the old group (52 vs. 22, p = 0.0004). Comorbidities were higher in old people than in young people according to CIRS. The clinical characteristics and type of orthopaedic surgery of the patients are reported in Table 1. All patients who underwent TKA suffered from severe knee osteoarthritis. The elapsed time from surgery to admission in rehabilitation ward was 7 ± 2 days. Low molecular weight heparin was administrated to all patients in the month after the surgery to prevent thrombotic and/or pulmonary embolic events. No difference in the number of subjects or the number of peri-operative blood units transfused was detected between the young and the old group. Three elderly subjects who underwent THA developed pulmonary infections and were not included in the analysis. Among these, one very old female subject died. The mean length of stay was 35.4 ± 8.4 days. The length of stay was longer in the old group than in the young group: 39.7 ± 7.1 and 29.1 ± 5.8, respectively (p < 0.0001).

**Table 1:** **Clinical characteristics of surgical orthopaedic subjects**

	**Patients**	**<= 75 y**	**>75 y**
**Number**	142	60	82
**Age**	77.2 (10.7)	66.9 (6.2)	84.7 (6.0)
**Male**	36	15	21
**Female**	106	45	61
**CIRS**	1.96 (1.07)	1.6 (1.2)	2.1 (0.9)
**Type of surgery**			
***THA***	76	25	51
***TKA***	26	17	9
***Internal fixation***	40	12	38
**Hip fracture**	74	22	52
**Hip arthritis**	42	22	20
**Knee arthritis**	26	17	9
**Time from surgery**	7 (2)		
**Length of stay**	35.7 (8.2)	29.5 (5.7)	39.7 (7.1)
Infections*/dead	3/1	-	3/1

Legend: CIRS = Cumulative Illness Rating Scale; THA = total hip arthroplasty; TKA = total knee arthroplasty. Standard deviation is reported in brackets; *pulmonary infections.

### Platelet count

At admission, the mean PLTC was 394.8 ± 147 × 100^9^/L (range 158–806 × 100^9^/L). Young people had significant higher values of PLTC than old subjects: 437.8 ± 145.9 and 362.9 ± 140.5, respectively (p = 0.004). Fifty-nine (42.4%) (42 female and 17 male) patients had PLTCs above the normal value (mean value 503.8 ± 117.8 × 100^9^/L, range 384–806 × 100^9^/L). Of these, 28 (20.1%; 21 female and seven male) had thrombocytosis with PLTCs above 500 × 100^9^/L, and 15 (10.7%; 11 female and four male) had a very high value above 600 × 100^9^/L. None had PLTCs under the normal laboratory value. Considering only those who had PLTC above 500 × 100^9^, no statistically significant difference was found between young and old subjects (603.4 ± 102.6 vs. 640.4 ± 84.4, p = 0.34) or between females and males (619.4 ± 100.3 vs. 608.4 ± 91.1, p = 0.80).

**Figure 2:**
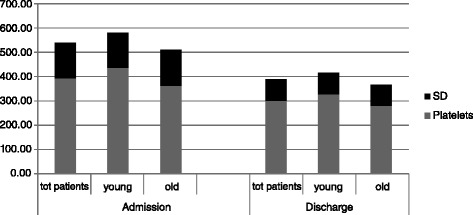
Platelet count in patients with major joint surgery.

At discharge, platelets levels had significantly decreased and the mean PLTC was within the normal laboratory value (300.4 ± 90.0 × 100^9^/L, range 104–599 × 100^9^/L, p < 0.0001), except for two patients who had low PLTC (104 and 122 × 100^9^/L). Platelets decreased more, but not significantly in young than in old people (−110.8 ± 131.4 vs. -81.9 ± 106.9, p = 0.18) and more in females than in males (−99.7 ± 118.3 vs. -76.9 ± 117.1, p = 0.35). PLTC remained above 400 × 100^9^/L in 17 (12.2%) subjects (443.3 ± 51.7 × 100^9^/L) (Figure 2). Of these, only three had high platelet values above 500 × 100^9^/L. One subject with HTA developed severe heparin-induced thrombocytopoenia requiring platelet transfusion and was transferred to the haematological ward. PLTC negatively correlated with the age at admission (r = −0.31, p = 0.0004) and at discharge (r = −0.25, p = 0.006). All patients with thrombocytosis underwent lower limb duplex scan ultrasonography within one week of PLTC detection (mean 5.1 ± 1, range 4–7 days). The examination was repeated in all patients in whom the PLTC was above 400 × 100^9^/L, at discharge. No subject with thrombocytosis and persistently elevated PLTC had lower limb DVT.

### Blood parameters

At admission, the mean values of ESR, fibrinogen, and D-dimers were elevated: 60.5 ± 25.4 (normal value: 2–15 mm); 506.1 ± 116.8 mg/dl (normal value: 200–450 mg/dl); and 580.7 ± 332.0 (normal value: 0–250 ng/ml); respectively. Young subjects had non-significantly higher values of ESR and fibrinogen than old people (61.5 ± 24.4 vs. 59.7 ± 26.3, p = 0.71; 520.6 ± 110.3 vs. 496.4 ± 121.1 mg/dl, p = 0.36, respectively). Conversely, old subjects had non-significantly higher mean value of D-dimers than young people: 614 ± 345.6 vs. 542.8 ± 317.6 (p = 0.2). Females had non-significantly different values of ESR and fibrinogen than males (60.8 ± 27.8 vs. 59.6 ± 17.2, p = 0.81; 491.8 ± 117.9 vs. 545.2 ± 106.5 mg/dl, p = 0.07, respectively). At discharge, ESR, fibrinogen and D-dimers mean values were significantly decreased: 38.1 ± 25.4, 393.3 ± 223.9 mg/dl, and 299.3 ± 168.2 ng/ml (p < 0.0001), respectively (Table 2). ESR decreased more, but not significantly so in young compared with old people (−37.1 ± 25.3 vs. -27.7 ± 26.7, p = 0.33) and in females compared with males (−34.0 ± 27.5 vs. -30.2 ± 16.5, p = 0.77). Fibrinogen decreased more, but not significantly so in young compared with old subjects (−215.8 ± 206.3 vs. -135.5 ± 103.9, p = 0.64) and in males compared with females (−320.0 ± 311.1 vs. -142.0 ± 109.7, p = 0.27). At admission, all subjects had mild anaemia as characterised by reduced RBC count and low Hb level (10.3 ± 2.4 gr/dl) that was significantly improved at discharge (11.2 ± 2 gr/dl. p < 0.0001). One female patient received a blood transfusion during her in-hospital rehabilitation stay, but she was not included in the analysis due to pulmonary infection.

**Table 2:** **Mean PLTC, blood parameters and functional outcome in patients with major orthopaedic surgery at admission to and discharge from the rehabilitation unit**

**Admission**				**Discharge**		
	**Patients**	**<75 y**	**>75 y**	**Patients**	**<75 y**	**>75 y**
**PLTC**	394.8* (147.0)	437.8*§ (145.9)	362.9* (140.5)	300.4 (90.0)	328.2$ (89.1)	281.0 (86)
**ESR**	60.5 (25.4)*	61.5 (24.4)*	59.7 (26.3)*	38.1 (25.4)	32.9 (26.5)	45.1 (23)
**Fibrinogen**	506.1* (116.8)	520.6* (110.2)	496.4* (121.1)	393.3 (223.9)	394.4 (264.4)	390.5 (145.0)
**D-dimers**	580.7* (332.0)	542.8* (317.6)	614* (345.6)	299.3 (168.2)	237.0 (137.4)	342.4 (179.0)
**RBC**	3490716.5 (477973.0)	3475555.6 (386374.9)	3501931.5 (538155.4)	3878871.6£ (435390.7)	3962687.5** (342441.9)	3812918.0 (489199.3)
**Hb**	10.3 (2.4)*	10.4 (2.9)*	10.2 (2)*	11.2 (2)	11.3 (0.8)	11.2 (2.6)
***Functional measures***						
**Gait**	1.3 (0.8)	1.8 (0.3)^	0.9 (0.8)	2.5 (0.7)**	2.9 (0.2)&	2.2 (0.8)**
**BS**	47.2 (22.5)	60.3 (15.3)§	36.0 (21.7)	82.7 (23.4)**	97 (6.9)§**	70.5 (25.6)**
**FIM**	66.5 (25.3)	82.5 (13.1)§	54.8 (25.6)	97.8 (29.4)**	116.4 (10.9)§**	83.6 (31.2)**

Legend: PLTC = platelet count × 100^9^/L; ESR = erythrocyte sedimentation rate; Hb = haemoglobin; RBC = red blood cells; BS = Barthel Scale; FIM = functional independence measure; standard deviation is reported in brackets; *p < 0.0001 (admission vs. discharge); £p = 0.025 (discharge vs. admission); **p = 0.0001 (discharge vs. admission); ^p < 0.0001 (young vs. old people); & p < 0.0001 (young vs. old people); §p = 0.004 (young compared to old); $p = 0.002 (young compared to old people).

### Functional outcome

On admission, the mean scores of the assessed functional measures were: 66.5 ± 25.3 and 47.2 ± 22.5 for FIM and BS, respectively. Old subjects had significantly poorer functionality than young ones according to FIM: 54.2 ± 25.6 vs. 82.5 ± 13.1 (p < 0.0001), and according to BS: 36.0 ± 21.7 vs. 60.3 ± 15.3 (p < 0.0001).

After rehabilitation treatment, functional improvement was observed: 97.8 ± 29.4 and 82.7 ± 23.4 (p < 0.0001) for FIM and BS, respectively. However, the young had significantly better outcomes than elder people in all functional assessment measures: 116.4 ± 10.9 vs. 83.6 ± 31.2, and 97.0 ± 6.9 vs. 70.5 ± 25.6 (p = 0.004) in the young and old groups for FIM and BS, respectively, at discharge (Table 2). Likewise, significant improvement in gait was observed at discharge compared to admission (2.5 ± 0.7 vs. 1.3 ± 0.8; p < 0.0001), but young people had better outcomes than old people: 2.9 ± 0.2 and 2.2 ± 0.8 (p < 0.0001), respectively (Table 2). Significant positive correlation between FIM and RBC was observed (p = 0.04) at discharge. Furthermore, BS and FIM positively correlated with Hb and albumin level (3.2 ± 0.3 and 3.6 ± 0.3 gr/dl, both on admission and discharge). Age was negatively correlated with platelet count at admission (r = −0.31, p = 0.0004) and at discharge (r = −0.25, p = 0.006). Age was negatively correlated with FIM and BS both at admission (r = −0.67, p < 0.0001; r = −0.63, p < 0.0001) and at discharge (r = −0.70, p < 0.0001; r = −0.66, p < 0.0001).

## Discussion

Change in platelet counts was a common phenomenon after major joint surgery, and elevated PLTC occurred in about half of the patients who were admitted to the rehabilitation centre. Thrombocytosis was not an uncommon event, since 20.1% of the sample had a PLTC above 500 × 100^9^/L, but this condition did not affect the functional outcome. Despite the risk of thromboembolism being a great concern in patients with thrombocytosis, no subject with elevated PLTC developed lower limb DVT.

Some of the most common causes of secondary thrombocytosis include soft-tissue, pulmonary and gastro-intestinal infections, as well as tissue damage and malignancy [1]. Furthermore, thrombocytosis is a common finding among patients with trauma, injuries and surgical interventions admitted to the ICU [6]. The significance of elevated PLTC in critically ill patients has not been clearly elucidated, although this sequela has generally been considered a benign finding. Only one study has been published reporting elevated PLTC or thrombocytosis after major orthopaedic surgery [10]. Surprisingly 163 (79.1%) of 206 subjects had elevated PLTC, with values from 400 to 450 × 100^9^/L. However, the authors focussed on extreme thrombocytosis, ascribing this finding to alcohol abuse and infections, and they were doubtful whether the finding was unique to orthopaedic surgery. Among secondary thrombocytosis, surgery including coronary artery by-pass, major abdominal surgery and especially splenectomy [3,1] are fairly common*.* Orthopaedic surgery was also reported as being associated with secondary thrombocytosis, but the finding concerned retrospective studies and thrombocytosis was generally attributed to other causes such as trauma and tissue damage [1]. To our knowledge, this is the first prospective study with a homogenous orthopaedic surgical sample and it demonstrates that high PLTCs occur in 42.4% of patients after major joint surgery. The role and significance elevated platelets might play in this clinical condition is unclear, though several hypotheses can be suggested. The major functions of platelets are to prevent acute blood loss and to repair vascular walls and adjacent tissues after a local injury. To accomplish this, platelets secrete many mediators and cytokines that stimulate tissue regeneration by cell proliferation, cell migration and angiogenesis [18]. It has been reported that more than 800 different proteins are released that act on different cell types, including osteoblasts [19], fibroblasts, chondrocytes, myocytes, and tendon cells [20-22]. Therefore, it could be postulated that major orthopaedic interventions activate physiological processes that heal the surgical wound, similar to the processes occurring during local injury. An increase in the amount of platelets may represent a component of that complex network of cells and inflammatory mediators that are activated following injury in order to repair and regenerate the damaged tissue. The observed increase in ESR, fibrinogen and D-dimer levels could also be considered as a further expression of an inflammatory response to repair the surgical wound. In the present study, elevated PLTC and inflammatory products were detected at about 7–12 days after surgery, when the patients were admitted to the rehabilitation unit. A similar finding was reported by Ziaja et al., who detected thrombocytosis two weeks after surgical intervention [11]. Likewise, in a study by Bunting et al., the highest PLTC occurred from nine to 22 days (mean 14 ± 3.0) after surgery [10]. Since platelets secrete many mediators and cytokines for at least seven days after a local injury [18], this time period could represent the peak of an adaptive response. In order to evaluate a potential age effect on platelet change, the enrolled subjects were divided in two groups according to an age cut-off of 75 years. Young people had significantly higher platelet counts than old people, at all time points. This finding could be explained by a better or more evident reactive-reparative response to the injury in young people compared with old people. It is also possible to consider that platelet change in old people is simply due to the progressive physiological multi-organ decline which occurs during ageing. A recent survey of PLTC in a large national cohort population found that the number of platelets decreases quickly in childhood, stabilises in adulthood, and further decreases with age [23]. The risk of thromboembolic events remains the greatest concern in patients with thrombocytosis, and subjects with elevated PLTC could be more likely to develop venous thromboembolism occurrence after major joint surgery. However, the relationship between elevated PLTC and these adverse events has not been established. One study by Salim et al. reported that there was a trend toward increased DVT, and a significantly higher rate of pulmonary embolism among patients developing thrombocytosis after severe trauma. However, this was not found in similar studies [3,6]. Despite this concern, no subject with thrombocytosis developed DVT in the present study. Global functional improvement was detected after rehabilitation treatment, but thrombocytosis did not affect the functional outcome. Similarly, a previous study reported that mortality risk was not correlated with PLTC in hip fracture patients [24]. On the other hand, univariate analysis of variables including age and blood parameters showed that these factors have an important role in functional recovery. Age was negatively correlated with FIM and BS both at admission and at discharge. Furthermore, BS and FIM were positively correlated with haemoglobin and albumin level. Similar findings have been reported in previous studies addressing the relationship between blood parameters, functional outcomes and mortality risk [12,25,26] in subjects undergoing joint surgery. The findings indicate that patients admitted to rehabilitation after major joint surgery have the potential for improvement in motor function and global functionality, but those with lower haemoglobin and albumin level and those who are older may expect longer hospital stays and poorer functional outcomes. Indeed, older people stayed in hospital longer and had significantly poorer outcomes than young people according to all of the functional assessment measures employed. Furthermore, significantly better gait recovery was observed in young compared with old people.

## Conclusion

Changes in platelet levels occurred after major joint surgery in patients admitted to a rehabilitation unit and elevated PLTC with thrombocytosis was not uncommon. This phenomenon may be a component of the underlying inflammatory process that acts to repair and heal the damaged tissue, but it does not promote DVT occurrence. Functional outcome was not affected by platelet changes or thrombocytosis. On the other hand, advanced age and low haemoglobin level predicted poor outcome.

## Abbreviations

DVT: Deep venous thrombosis
ICU: Intensive care unit
THA: Total hip arthroplasty
TKA: Total knee arthroplasty
CIRS: Cumulative Illness Rating Scale
BS: Barthel scale
FIM: Functional Independence Measure
PLTC: Platelet count
ERS: Erythrocyte sedimentation rate
RBC: Red blood cells
Hb: Haemoglobin
